# Preparedness of the Local Population for the Uptake of Artificial Intelligence and Digital One Health for Home Healthcare of Emerging and Reemerging Infectious Diseases in Southwest and Littoral Regions of Cameroon

**DOI:** 10.1155/jotm/8896234

**Published:** 2025-08-19

**Authors:** Ettah Agnes Asonganyi, Elvis Asangbeng Tanue, Ginyu Innocentia Kwalar, Odette Dzemo Kibu, Moise Ondua, Maurice Marcel Sandeu, Patrick Jolly Ngono Ema, Denis Nkweteyim, Madeleine L. Nyamsi, Peter L. Achankeng, Christian Tchapga, Justine Ayuk, Gregory Eddie Halle-Ekane, Jude Dzevela Kong, Dickson Shey Nsagha

**Affiliations:** ^1^Department of Public Health and Hygiene, Faculty of Health Sciences, University of Buea, P.O Box 63, Buea, Cameroon; ^2^DigiCare Cameroon Consortium, University of Buea, South West Region, Buea, Cameroon; ^3^Department of Microbiology and Infectious Diseases, School of Veterinary Medicine and Sciences, University of Ngaoundéré, Po Box 454, Ngaoundéré, Cameroon; ^4^Department of Genetics and Biostatistics, School of Veterinary Medicine and Sciences, University of Ngaoundéré, Po Box 454, Ngaoundéré, Cameroon; ^5^Department of Computer Science, Faculty of Science, University of Buea, P.O Box 63, Buea, Cameroon; ^6^Department of Women and Gender Studies, Faculty of Social and Management Sciences, University of Buea, P.O Box 63, Buea, Cameroon; ^7^Department of Obstetrics and Gynaecology, Faculty of Health Sciences, University of Buea, P.O Box 63, Buea, Cameroon; ^8^Department of Mathematics & Statistics, York University, Toronto, Canada

**Keywords:** artificial intelligence, Digital One health, emerging and reemerging infectious diseases, home health care, preparedness

## Abstract

**Background:** Rapid digital responses to pandemics highlight advancements in healthcare, data sharing, and artificial intelligence (AI). While AI has driven progress in precision medicine, drug discovery, and vaccine development, its application to emerging and reemerging infectious diseases (ERIDs) remains underexplored, presenting critical challenges in addressing future health threats.

**Objectives:** The study evaluated knowledge of ERIDs, AI, and Digital One Health (DOH) technologies, examined preparedness for their adoption in home healthcare, and identified factors influencing readiness to utilize these technologies in selected health districts of Cameroon.

**Methods:** A cross-sectional study assessed the preparedness of communities in Buea, Limbe, Bonassama, and New-Bell Health Districts to adopt AI and DOH technologies from April to May 2024. Systematic random sampling included 33 communities, with data collected using face-to-face structured questionnaires. Analysis using SPSS Version 26 involved descriptive statistics and logistic regression, with statistical significance set at *p* < 0.05 and a 95% confidence interval to identify key associations.

**Results:** Among 1625 participants, only 280 (17.2%) had good knowledge of ERIDs, with COVID-19 (68.8%) and cholera (94.5%) being the most recognized examples. Knowledge of AI and DOH technologies was poor, with only 166 (10.2%) demonstrating accurate understanding. Early disease detection emerged as a critical application of AI for ERID control. Preparedness to adopt AI and DOH technologies was reported by 941 (57.9%), with 64.5% comfortable with AI-generated interpretations and willing to use digital health tools during ERID outbreaks. Factors independently associated with preparedness included being a student (AOR = 2.678; 95% CI: 1.744–4.113; *p* < 0.001), good knowledge of AI and DOH (AOR = 7.141; 95% CI: 4.192–12.162; *p* < 0.001), and prior training on AI and digital health (AOR = 3.081; 95% CI: 2.272–4.179; *p* < 0.001).

**Conclusion:** The study revealed insufficient knowledge of ERIDs, AI, and DOH but high preparedness to adopt these technologies for home care. Enhanced educational campaigns are recommended to improve community understanding and effective utilization of AI and DOH for controlling ERIDs.

## 1. Background

One Health (OH) is an integrated, unifying approach that aims to sustainably balance and optimize the health of humans, animals, and ecosystems by integrating these fields, rather than keeping them separate [1]. It unites various fields of study and involves participation from all parts of society to foster cooperation and advance human, animal, and ecosystem well-being and addresses health and environmental challenges [2].

Emerging infectious diseases and the rise of zoonotic pathogens, such as human immunodeficiency virus (HIV), directly contribute to an estimated 15 million deaths worldwide each year [3]. Estimates suggest that 60% of current human infections and 70% of newly emerging infectious diseases in humans originated in animal populations [4]. Close contact among humans, plants, animals, and the environment provides an increased risk of disease transmission [5]. Emerging and reemerging infectious diseases (ERIDs) are susceptible to explosive outbreaks and constitute deadly epidemic threats that could rapidly reach pandemic proportions, affecting people's lives [5]. In a world of heightened interaction, the production of animal-derived food for human consumption, expanded transportation usage, and increased cross-border mobility of individuals collectively influence the occurrence and spread of infectious diseases either directly or indirectly [6]. Successfully controlling infectious diseases requires measures grounded in comprehending disease transmission routes, enhancing surveillance systems, and implementing interventions [5]. These achievements can be realized through the application of the OH approach [5]. Although OH aims to enhance the well-being of humans, other animals, and the interconnected ecosystem, its present application predominantly emphasizes collaborative efforts among individuals rather than data integration streams. It is understandable that such data incorporation is constrained by ethical, legal, political, and social considerations [7]. The COVID-19 pandemic starkly emphasized the necessity of data, information, and knowledge exchange among governments, healthcare systems, the public, and other pertinent stakeholders, and this can be facilitated by technology. Even with the efforts of high-income countries to integrate data with robust surveillance systems, full integration is still hampered by legal issues regarding data sharing, and this is where the advantage of Digital One Health (DOH) becomes evident as it explicitly acknowledges and tackles ethical and legal constraints within its framework [7]. Transforming OH through digitalization involves utilizing digital technologies such as artificial intelligence (AI), big data, and related digital innovations to enhance the abilities in addressing the increasing environmental challenges and associated risks to human, animal, and plant health [8]. The swift digital responses to pandemics have underscored rapid advancements in certain areas such as precision medicine, drug discovery, and vaccine development. However, numerous substantial challenges persist, particularly in areas such as service delivery, data sharing, and the implementation of AI [4]. AI methods have been utilized in health areas such as precision medicine, drug exploration, and vaccine creation. Nonetheless, relatively less focus has been given to the application of AI for ERIDs [9]. Research has shown that utilizing AI in the realm of OH demonstrates excellent potential for enhancing disease diagnosis, selecting appropriate treatment, and conducting clinical laboratory testing [10, 11]. AI plays a pivotal role by offering advanced analytics, predictive modeling, and decision support, thereby facilitating improved disease surveillance, outbreak prediction, and resource allocation vital for addressing complex health concerns [12]. Despite the benefits of using AI to improve healthcare, community preparedness in the uptake of AI and DOH technologies is still an essential aspect in its initiation, implementation, and utilization. Thus, the aim of the study was to assess community's knowledge on ERIDs, AI, and DOH technologies, determine the level of preparedness, and identify factors influencing preparedness to uptake these technologies for home care.

## 2. Methods

### 2.1. Study Design

This was a community-based, cross-sectional, face-to-face interview-administered survey conducted in 33 communities randomly selected from the Buea, Limbe, Bonassama, and New-Bell Health Districts from April to May 2024. Community members aged 18 years and above who lived in communities in Buea, Limbe, Bonassama, and New-Bell Health Districts were recruited. The level of preparedness to utilize these technologies for home care of ERIDs was the response variable, while sociodemographic characteristics and knowledge on these technologies were exposure variables.

### 2.2. Study Participants and Setting

A total of 1625 community members aged 18 years and above living in 33 communities in Buea, Limbe, Bonassama, and New-Bell Health Districts participated in this survey. The Buea Health District is found in the Southwest Region of Cameroon with an estimated population of about 151,263 inhabitants [13]. It is one among the two Anglophone (English-speaking) towns in Cameroon. It is made up of 7 health areas: Bova, Buea Town, Bokwango, Tole, Buea Road, Muea, and Molyko health areas within which there are many different communities. The Limbe Health District is also found in the Southwest Region of Cameroon with an estimated population of about 72,106 inhabitants [14]. It is covering a surface area of 674 square km. It is divided into eight health areas: Bota, Bojongo, Idenau, Moliwe, Mabeta, Limbe Sea Port, Zone II, and Batoke health areas within which there are many different communities made up of people with different cultures. The Bonassama and New-Bell Health Districts in the Littoral Region of Cameroon have health facilities which include a combination of public health institutions, faith-based organizations, and private hospitals, collectively contributing to the region's healthcare landscape and service provision. In terms of healthcare technological infrastructure, it is equipped with a network of health districts and facilities, serving the healthcare needs of the local population.

### 2.3. Sample Size Determination

The sample size for this study was determined using the sample size calculation for a frequency in a population formula given by

(1) $$\begin{array}{c} n=\frac{\left[\text{DEFF}*N*P\left(1-P\right)\right]}{\left[d^{2}/z^{2}*\left(N-1\right)+P\left(1-P\right)\right]}, \end{array}$$

where *n* = sample size for the study, *P* = prevalence/proportion = 50% = 0.5, *d* = margin of error = 5% = 0.005, *Z* = 95% confidence interval (CI) *z*-score = 1.96, DEFF = design effect = 4, and *N* = hypothesized population of Southwest and Littoral Regions of Cameroon = 4,908,000 [15].

Therefore,

(2) $$\begin{array}{c} n=\frac{\left[4*4,908,000*0.5\left(1-0.5\right)\right]}{\left[0.005^{2}/1.96^{2}*\left(4,908,000-1\right)+0.5\left(1-0.5\right)\right]}=1,537. \end{array}$$

After adding a 5% attrition = [5 × 1537]/100 = 77 participants, a final sample size of 1614 community members was obtained.

### 2.4. Sampling Method

**Figure 1:**
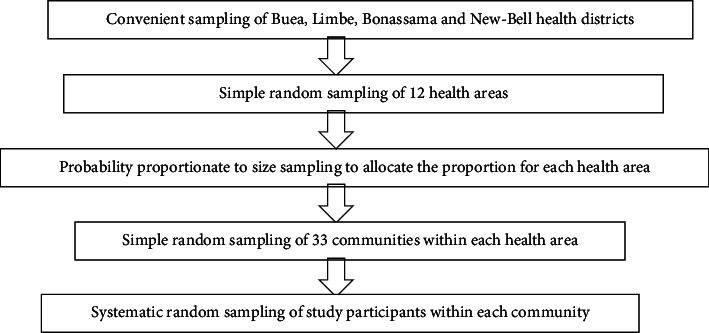
Multistage sampling of study community members.

Figure 1 shows a multistage random sampling method that was used to select community members aged 18 years and above who resided in communities in Buea, Limbe, Bonassama, and New-Bell Health Districts. In order to fulfill the sample size criteria, the sampling process was performed in five steps: In Step 1, two health districts (Buea and Limbe) in the Southwest Region and two health districts (Bonassama and New Bell) in the Littoral Region were conveniently selected. In Step 2, a combined list of 36 health areas from all four health districts was compiled. From this list, 12 health areas were randomly selected; specifically, 3 were selected from each district. The selected areas included Molyko, Muea, Buea Road, Bota, Zone II, Sea-Port, Bilingue, Grand-Hangar, Sodiko, Nkololoun, Nkolmitag, and Sebenjongo. In Step 3, a list of 215 communities from all the 12 health areas combined was made, whereby a random selection of 33 communities (Paramount, Wonjomba, Checkpoint, Kombo, Bolifamba, Wonya Mavio, Mile 17, Federal Quarters, Long Street, Great Soppo Native, Lower Bonduma, Wotolo, Church Street, Down Beach, Mabeta Layout, Livanda North, Livanda South, Towe, Limbe Camp, Mokindi, Middle farms, Bloc 2-Grand Hangar, Bloc 3-Grand Hangar, Bloc 8-Sodiko, Bloc 9-Sodiko, Bloc 2-Bilingue, Bloc 3-Bilingue, Denier Porto, Tracafric, New-Bell Gare, New-Bell Center, Bloc A-Nkolmitag, and Bloc B- Nkolmitag) was made from the list. In Step 4, within each selected health area, a probability proportionate to size sampling was used to get the number of participants that were selected from communities in each health area. And finally, a random selection of community members was done using the systematic sampling technique, with the skip interval by dividing the total number of community members in each selected community in the health area by the desired sample size from each community. The systematic sampling started by randomly selecting a number between 1 and the skip interval. The first individual selected was the person corresponding to the randomly chosen number, and subsequent individuals were selected at regular intervals according to the skip interval, limiting participation to a maximum of 2 members per household. This sampling technique was done to ensure that each community member in the selected health area has an equal chance of being included in the study.

### 2.5. Data Collection

Three graduate students were employed as research assistants and received training on the project, data collection procedures, and research ethics. The initial study's questionnaires were pretested, and any necessary changes were made before data collection. Questions were mostly asked in French, English, and Pidgin-English at the convenience of the participants. The questionnaires were administered using face-to-face interviews, and all data were gathered using the KoboCollect application. Community members were met at their households, and a systematic random sampling technique was used to select community members using the *k*th interval while limiting participation to a maximum of 2 members per household. Community members were asked a series of questions some of which included their awareness and knowledge of ERIDs, AI, and DOH technologies and also questions on how prepared they think they were to utilize these technologies to receive healthcare at home. Specifically, participants were asked close questions that provided answer options that participants could select from such as “if they had heard about ERIDs, if they knew what they were, if they could give examples, if they had heard about AI and DOH technologies, and if they knew of the importance of using these technologies to receive healthcare at the comfort of their homes.”

### 2.6. Outcome Measures

The questionnaire consisted of four sections: sociodemographics, knowledge on ERIDs, knowledge on AI and DOH, preparedness to utilize AI and DOH technologies, and factors influencing preparedness to use AI and DOH technologies. The questionnaire had 38 questions: 12 on knowledge, 9 on the level of preparedness, 7 on factors influencing the level of preparedness to use these technologies, and 10 on sociodemographics. Some questions were asked on a yes/no basis with an additional “I don't know option,” and some were with structured closed-ended which presented respondents with a predefined set of answers to choose from.

### 2.7. Statistical Analysis

The Statistical Package for Social Sciences (SPSS) Version 26.0 was used to analyze all data. Frequencies and percentages were calculated for categorical variables, and for continuous variables, means and standard deviations were reported. A score of 1 was attributed to a correct answer and 0 to a wrong answer for knowledge and preparedness. The overall scores of each individual were used to obtain mean scores for knowledge and preparedness thereafter which 60% of the total highest score was used as a cut-off point in which those with scores lower than 60% were considered to have poor knowledge and low preparedness and those equal to or higher than 60% were considered to have good knowledge and high preparedness. This 60% cutoff point for defining knowledge and preparedness was based on a previous study conducted in China whereby the bloom cutoff was used for defining knowledge and practices [16]. Frequencies of good knowledge and high preparedness were described. A binary logistic regression analysis was used to screen factors for the multivariate analysis. Multiple logistic regression analysis using all sociodemographic variables, knowledge on AI and DOH, and other factors (personal, economic, and social) as independent variables, and level of preparedness as the outcome variable was conducted to identify factors significantly associated with preparedness to utilize AI and DOH technologies to receive healthcare at home. Equally, a multiple logistic regression analysis using all sociodemographic variables as independent variables and the knowledge score as the outcome variable was conducted to identify factors significantly influencing knowledge on AI and DOH technologies.

Factors were chosen using a backward stepwise approach. Unstandardized regression coefficients (β) and odds ratios (OR) with 95% CIs were employed to assess the relationships between variables and knowledge and level of preparedness, with a statistical significance threshold set at *p* < 0.005.

### 2.8. Ethical Considerations

All procedures conducted in this study adhered to the ethical standards set by the Institutional Review Board (IRB) of the Faculty of Health Sciences, University of Buea. The research protocol received approval from the IRB, University of Buea (2024/2344-01/UB/SG/IRB/FHS). Administrative authorizations were obtained from the Department of Public Health and Hygiene, Faculty of Health Sciences, Southwest Regional Delegation of Public Health and Littoral Regional Delegation of Public Health, District Health Services of Buea, Limbe, Bonassama, and New-Bell, and from the 12 health areas before data collection. Local authorizations were obtained from the heads of selected communities. Informed consent was obtained from each community member and assent equally sought from parents or guardians of community members less than 21 years. Community members who agreed to participate in the study were required to sign on the informed consent document, and this was done only after discussing with individual participants the purpose of the study, their rights with respect to the study, and their participation in the interview. Anonymity was respected, as the names of the participants were not mentioned in the data collection process.

## 3. Results

### 3.1. Sociodemographic Characteristics

A total of 1625 community members completed the survey with an average age of 34.58 ± 12.15 years. The sociodemographic details of the study participants are summarized in Table 1. Among the respondents, 54.6% were female and 49.3% reported secondary education as their highest educational level. About main occupation, 31.2% were self-employed and 55.8% had an estimated monthly income of below 50,000 FCFA. With regard to technology, 86.2% of the respondents owned a smartphone, and out of this, a total of 85.7% had access to internet connectivity. Overall, 28.1% resided in the Buea Health District.

### 3.2. Knowledge on ERIDs Among Community Members in the Buea, Limbe, Bonassama, and New-Bell Health Districts

Table 2 shows the knowledge of community members on ERIDs. The results showed that 280 (17.2%) community members had correct knowledge, while 1345 (82.8%) had incorrect knowledge on ERIDs. A limited number of participants had heard about emerging 610 (37.5%) and reemerging 677 (41.7%) infectious diseases, and among those who had heard of ERIDs, a greater number of participants indicated that COVID-19 833 (68.8%) and cholera 945 (73.1%) were the main examples of ERIDs, respectively. Poor hygiene and sanitation 1155 (79.1%) and global climate change 614 (42.0%) were revealed to be the major causes of ERIDs with which getting vaccinated 1092 (73.7%), cleaning and disinfecting used surfaces 775 (52.3%), and handling food safe 757 (51.1%) were the major preventive measures used for the control of ERIDs as reported by the participants.

### 3.3. Knowledge on AI and DOH Technologies Among Community Members in the Buea, Limbe, Bonassama, and New-Bell Health Districts

Table 3 shows the knowledge of community members on AI and DOH technologies. The results showed that 166 (10.2%) community members had good knowledge, while 1459 (89.8%) had poor knowledge on AI and DOH. Less than half of the participants had heard about AI 518 (31.9%), OH 253 (15.6%), and DOH 235 (14.5%). Among the study participants who had heard of AI and DOH, 407 (81.7%) defined AI as the use of technologies that empowers machines to carry out healthcare duties that demand human intelligence and 63 (3.9%) defined DOH as the use of digital technologies and data to better understand how human, animal, and plant health are interconnected. A vast number of community members did agree that early disease detection 733 (61.1%), early diagnosis and treatment 512 (42.7%), and better response to tracking of infectious diseases 711 (59.3%) were the importance of using AI for receiving healthcare. Additionally, wearable smart watches 799 (68.7%) were reported as an AI tool in healthcare, while Waspito 176 (38.5%) and period calendar (PC) 285 (62.4%) were reported as examples of digital health technologies.

### 3.4. Association of Sociodemographic Variables and Knowledge on AI and DOH Among Community Members in the Buea, Limbe, Bonassama, and New-Bell Health Districts

Table 4 shows adjusted models for the association between sociodemographic characteristics and knowledge on AI and DOH technologies. In multivariate logistic regression analysis, community members from the Buea Health District were more likely (AOR = 3.724; 95% CI: 2.176–6.371; *p* = 0.001) to have good knowledge on AI and DOH technologies compared to those from the New-Bell Health District. Male participants were more likely to have good knowledge on AI and DOH (AOR = 1.603, 95% CI: 1.129–2.276; *p* = 0.008) than female participants. Additionally, the odds of participants with primary level of education having good knowledge on AI and DOH were twice as much as the (AOR = 1.797 95% CI: 1.100–2.936; *p* = 0.019) odds of participants with tertiary level of education having good knowledge on AI and DOH. The odds of students having good knowledge on AI and DOH (AOR = 2.741, 95% CI: 1.483–5.066; *p* = 0.001) were more than 2 times as much as the odds of participants with no job having good knowledge on AI and DOH.

### 3.5. Preparedness of Community Members in the Buea, Limbe, Bonassama, and New-Bell Health Districts to Utilize AI and DOH for Home Healthcare Delivery for the Control of ERIDs

The mean score of preparedness to utilize AI and DOH technologies was 5.51 ± 2.95. The results showed that 941 (57.9%) community members had high preparedness level, while 684 (42.1%) had low preparedness level to utilize AI and DOH technologies to receive healthcare at home. Even though less than half of the study participants were prepared to use DOH technologies 524 (32.2%) and AI 611 (37.6%) for home healthcare, a greater number were confident about embracing these new technologies into their setting 667 (41.0%). A majority of participants reported having not attended any previous training on AI and DOH tools 1276 (78.5%) for healthcare but were willing to participate in training programs if organized 1109 (68.2%). Majority of the participants were comfortable with AI interpretations 1048 (64.5%), willing to recommend AI to others 1122 (69.0%), and to receive care from healthcare providers through AI 1101 (67.8%) as seen in Table 5.

### 3.6. Sociodemographic Factors Independently Associated With Preparedness to Use AI and DOH Technologies for Home Healthcare for the Control of ERIDs Among Community Members in the Buea, Limbe, Bonassama, and New-Bell Health Districts

Table 6 shows adjusted models for the association between sociodemographic characteristics and preparedness to utilize AI and DOH technologies, using multivariate logistic regression with a *p* value of < 0.05 as the cutoff value for statistical significance. Two factors (level of education and main occupation) were found to be significantly associated with level of preparedness to utilize AI and DOH.

Participants with informal (AOR = 0.476; 95% CI: 0.295–0.767; *p* = 0.002) and secondary (AOR = 0.703; 95% CI: 0.538–0.919; *p* = 0.010) education levels were significantly less likely to be prepared to use AI and DOH for home healthcare compared to those with tertiary education. Additionally, students (AOR = 2.678; 95% CI: 1.744–4.113; *p* ≤ 0.001) were significantly more likely to be highly prepared to utilize AI and DOH compared to individuals with no job.

### 3.7. Other Factors Independently Associated With Preparedness to Utilize AI and DOH for Home Healthcare for the Control of ERIDs Among Community Members in the Buea, Limbe, Bonassama, and New-Bell Health Districts

Table 7 shows adjusted models for the association between other factors (personal, economic, and social) and preparedness to utilize AI and DOH technologies to receive home healthcare. In multivariate logistic regression using a *p* value < 0.05 as the cutoff value for statistical significance, 8 factors were found to be significantly associated with level of preparedness to utilize AI and DOH. The study revealed that participants with good knowledge had 7.141 (95% CI: 4.192–12.162; *p* ≤ 0.001) times higher odds of being prepared to use AI and DOH technologies compared to those with poor knowledge being prepared to use these technologies. Worry about a lack of human supervision in healthcare decision-making was negatively associated with preparedness, whereby the odds of being prepared to utilize AI and DOH were significantly lower among those who worried about this issue (AOR = 0.783, 95% CI: 0.621–0.988; *p* = 0.039). In addition, those who perceived a lack of access to AI technology as a barrier had 1.313 (95% CI: 1.032–1.672; *p* = 0.027) times higher odds of being prepared to use AI and DOH compared to those who did not. Believing that subsidizing the cost of AI and DOH (AOR = 1.002; 95% CI: 1.002–1.628; *p* = 0.048) and utilizing it in collaboration with healthcare providers (AOR = 1.818; 95% CI: 1.404–2.354, *p* ≤ 0.001) could make AI accessible was significantly associated with high preparedness to use these technologies. It was also revealed that participants who believed that healthcare systems were willing to integrate and utilize AI had 3.382 (95% CI: 2.67–4.284; *p* ≤ 0.001) times higher odds of being prepared to utilize AI. Finally, participants who had received training on AI and DHTs had 3.018 (95% CI: 2.272–4.179, *p* ≤ 0.001) times higher odds of being prepared to use these technologies.

## 4. Discussion

The main finding of the present study can be summarized as follows: Overall, the study revealed only 280 (17.2%) having good knowledge of ERIDs. Knowledge of AI and DOH technologies was poor, with only 166 (10.2%) demonstrating accurate understanding. However, preparedness to adopt AI and DOH technologies was reported by 941 (57.9%), with a greater proportion of the population being comfortable with AI-generated interpretations and willing to use digital health tools during ERID outbreaks. Notably being a student, having good knowledge on AI and DOH, and having prior training on AI and digital health were some factors found to be independently associated with preparedness to utilize AI and DOH technologies.

The findings revealed that majority of the study participants had never heard about emerging 1015 (62.5%) and reemerging 9948 (58.3%) infectious diseases, and this could be due to the fact that most participants had a secondary level of education and were not healthcare providers to understand the terms clearly, and among those who had heard about ERIDs, a majority could clearly state that diseases such as COVID-19 and cholera were main examples of ERIDs, respectively, and majority were knowledgeable that these diseases could be mostly caused by poor hygiene and sanitation. These findings were higher than in a study carried out in Southern China where 32.05% of participants had never heard about ERIDs such as MERS, ZIKA, Ebola, and plagues [17].

Overall, only 280 (17.2%) community members had good knowledge on ERIDs, and this finding was different from a study carried out in Southern China where knowledge rates about ERIDs such as Ebola and plague were over 50%, and this discrepancy might be explained due to the fact that the China government had initiated national emerging response to Ebola virus through measures including health information reasons for a high knowledge rate of emerging infectious diseases [17] as compared to our study.

In the study, less than half of the participants had heard about AI 518 (31.9%), OH 253 (15.6%), and DOH 235 (14.5%) even though only a fewer number of this could tell correctly what AI (407, 31.9%), OH (67, 4.1%), and DOH (63, 3.9%) really meant. This findings were different from findings from a study carried out in Germany, where more than 90% had already heard about AI though only 24% reported to have good or expert knowledge on AI, and this could be because their study assessed knowledge among patients who might have in one or another way heard about AI from their healthcare providers in their health facility [18]; however, this finding was contrary to a study conducted in Turkiye where 63.5% of physicians had not heard about OH [19].

This study found that a limited number of community members had good knowledge 166 (10.2%) on AI and DOH, and this finding was different from a study carried out in Germany where 24% of participants reported to have good or expert knowledge on AI, and this could be because about 25% of the respondents were from the medical or health professional backgrounds [18] and also different from a study carried out in the United States, where 31.0% of their participants had at least basic knowledge on OH [20].

Our study revealed a significant association between sex, main occupation, health district, and the level of education. Male participants were more likely to have good knowledge on AI and DOH compared to female participants, and this could be because males are somehow more exposed to and interested in technology in our setting than females, and this was similar to a study carried out in Vietnam where male students had higher knowledge on AI compared to female students [21]. Students were also more likely to demonstrate good knowledge on AI and DOH compared to other occupations, and this could be due to the fact that students could have been more inquisitive to acquire more knowledge, and they are more exposed to the possible sources of getting information. This finding was similar to a study carried out in Syria where postgraduate students were more knowledgeable about AI compared to professors and other professions [22]. There was also a significant good knowledge on AI and DOH among participants from the Buea Health District, and this could be attributed to the fact that it is made up of younger people who are mostly students with more exposure to sources of information about technological advancements than the other districts.

This study revealed high level of preparedness (57.9%) to use AI and DHTs among community members with a mean preparedness score of 5.51 though a majority still reported not being confident to embracing new technologies such as the use of AI tools into their setting even if made available and accessible to them since they were not comfortable with AI interpretations of medical test results and provision of medical care and advice, and this could be as a result of the fact that there was poor knowledge about AI and DOH technologies and its importance to healthcare outcomes. This is in accordance with a study carried out in Catalonia, where healthcare workers were also prepared to utilize AI, and this similarity could be because participants from both studies considered AI as an ally for improving quality of care and not being so concerned about the possibility of being replaced by AI in future [23].

There was significant interdependence between the level of education, main occupation, and the level of preparedness to utilize AI and DOH. Community members with informal and secondary levels of education were less likely to be prepared to utilize AI and DOH technologies compared to those at tertiary levels, and this could be because those at tertiary levels might have been more exposed to more educational and training programs which might have in one way or another increased the tendency of knowing and hearing about AI. However, there was no significant association between age and the level of preparedness to use AI and DOH technologies, and this was similar to a study carried out in Malaysia where there was no significant relationship between age and the cognitive domain of AI readiness [24] because all respondents were comfortable embracing and interacting with new technologies. Furthermore, students were found to be more likely prepared to utilize AI and DOH technologies than community members with different occupations, and this could be due to the fact that most students have some level of knowledge about AI and its importance and also due to the fact that AI is thought to help in assisting in studies; however, this was contrary to a study in Ontario, where most students (79%) claimed that their medical education was inadequately preparing them to work alongside AI tools or applications as they agreed that more preparations were needed in the medical program to increase their AI readiness level [25].

In addition, having good knowledge on AI and DOH technologies was found to be significantly associated with high preparedness to utilize AI and DOH technologies. Community members with good knowledge on AI and DOH technologies were 7.141 times highly prepared to use AI and DOH technologies than community members with poor knowledge, and this could be because knowing basic information about AI and its importance in improving quality healthcare can in one way increase community members' willingness to embrace these technologies and adopt and utilize them. This finding was contrary to a study in Malaysia, where there was no significant association between awareness of AI use in the medical field and medical readiness [24]. The study also showed a significant association between previous training and preparedness to utilize AI and DOH technologies. Community members who agreed to have received some training on the use of AI and other digital health technologies such as mobile health apps were found to be more likely prepared to utilize AI than those who had not received any previous training, and this finding was similar to a study carried out in UK, where medical students who received previous training in AI were more positive toward AI-medical readiness and were more prepared to collaborate with the use of AI technology [26], and this similarity could be because training sessions are important facilitative factors that can enhance preparedness to use these technologies.

Also, healthcare systems being willing to utilize AI and DHTs were also found to be a significant factor in increasing preparedness of community members to adopt and utilize AI for the control of ERIDs, and this association could be due to the fact that if healthcare settings are at the first front to adopting and utilizing AI and DOH technologies, it might therefore serve as an encouragement to community members to overcome their fears of the disadvantages or worries and concerns about AI and hence increase trust in these technologies and thus enhance preparedness to use these technologies.

## 5. Conclusion

Overall, the study revealed that a limited number of community members demonstrated good knowledge on ERIDs (17.2%), AI, and DOH (10.2%). Although most participants had poor knowledge on AI and DOH, most of them were prepared (57.9%) to use AI and DOH technologies to receive care at home for the control of ERIDs. Having informal and secondary levels of education, being a student, having good knowledge on AI and DOH, having previous training on AI and digital health technologies, access to AI technologies, willingness of healthcare systems to integrate AI, concerns such as AI replacing healthcare workers, subsidizing cost of AI, and using AI in collaboration with healthcare providers were found to be independently associated with preparedness to use AI and DOH for receiving healthcare at home for the control of ERIDs. More sensitization campaigns should be done in communities to enhance knowledge on ERIDs, AI, and DOH technologies. Also, community members should engage in training programs, seminars, and workshops to improve on skills needed to utilize and maintain these technologies as this will help enhance preparedness to adopt and use these technologies.

## Figures and Tables

**Table 1:** Sociodemographic characteristics of community members in the Buea, Limbe, Bonassama, and New-Bell Health Districts of Cameroon.

Variables	Category	No. (%)
Health district	Buea	457 (28.1)
	Limbe	442 (27.2)
	Bonassama	348 (21.4)
	New-Bell	378 (23.3)
	Total	1625 (100.0)

Age group (in years)	18–29	686 (42.2)
	30–39	457 (28.1)
	40–49	244 (15.0)
	50–59	176 (10.8)
	≥ 60	62 (3.8)
	Total	1625 (100.0)

Sex	Female	887 (54.6)
	Male	738 (45.4)
	Total	1625 (100.0)

Level of education	No formal	101 (6.2)
	Primary	305 (18.8)
	Secondary	801 (49.3)
	Tertiary	418 (25.7)
	Total	1625 (100.0)

Main occupation	Employed in public sector	107 (6.6)
	Employed in private sector	221 (13.6)
	Self-employed	713 (43.9)
	Religious leader	16 (1.0)
	Unemployed	119 (7.3)
	Student	282 (17.4)
	Jobless	167 (10.3)
	Total	1625 (100.0)

Smartphone ownership	No	224 (13.8)
	Yes	1401 (86.2)
	Total	1625 (100.0)

Access to internet connectivity	No	232 (14.3)
	Yes	1393 (85.7)
	Total	1625 (100.0)

Estimated monthly income (FCFA)	Below 50,000	906 (55.8)
	50,000–100,000	481 (29.6)
	101,000–150,000	102 (6.3)
	Above 150,000	136 (8.4)
	Total	1625 (100.0)

**Table 2:** Knowledge on emerging and reemerging infectious diseases among community members in the Buea, Limbe, Bonassama, and New-Bell Health Districts of Cameroon.

Variables	Category	No. (%)
Heard of EIDs	No	1015 (62.5)
	Yes	610 (37.5)

Definition of EIDs	Diseases that are new or spreading quickly in a population which can be caused by specific types of germs	432 (26.6)
	Diseases that are not caused by infections but develop and can lead to health problems	102 (6.3)
	Group of diseases where the body's cells start growing out of control	21 (1.3)

Examples of EIDs	Diabetes	230 (19.0)
	COVID-19	833 (68.8)
	Monkey pox	271 (22.4)
	Cancer	177 (14.6)
	HIV	319 (26.3)
	Hepatitis B	207 (17.1)
	Ebola virus	322 (26.6)

Heard about RIDs	No	948 (58.3)
	Yes	677 (41.7)

Definition of RIDs	Diseases caused by exposure to chemical toxins	79 (4.9)
	Diseases caused by growth of body's cells	115 (7.1)
	Diseases that were once a big issue but then got better, only to come back and affect a lot of people again	378 (23.3)

Examples of RIDs	Tuberculosis	312 (24.1)
	Hypertension	157 (12.2)
	Chlamydia	161 (12.5)
	Cholera	945 (94.5)
	Malaria	596 (46.1)
	Poliomyelitis	323 (11.8)
	Measles	248 (19.2)

Causes of ERIDs	Population growth/density	576 (39.4)
	Poor hygiene/sanitation	1155 (79.1)
	Physical activity	168 (11.5)
	Global climate change	614 (42.0)
	Poor food distribution/poor vaccination practices	583 (39.9)
	Drug/alcohol abuse	219 (15.0)
	Witchcraft	83 (95.7)

Prevention of ERIDs	Get vaccinated	1092 (73.7)
	Handle and prepare food safely	757 (51.1)
	Avoid unvaccinated animals	619 (41.8)
	Engage in physical activity	250 (16.9)
	Clean and disinfect surfaces	775 (52.3)
	Wash hands often	628 (42.4)
	Alcohol/drug intake	42 (2.8)

Overall knowledge level	Good knowledge	280 (17.2%)
	Poor knowledge	1345 (82.8%)

Abbreviations: EIDs, emerging infectious diseases; ERIDs, emerging and reemerging infectious diseases; RIDs, reemerging infectious diseases.

**Table 3:** Knowledge on artificial intelligence and Digital One Health technologies among community members in the Buea, Limbe, Bonassama, and New-Bell Health Districts of Cameroon.

Variables	Category	No. (%)
Heard about AI	No	1107 (68.1)
	Yes	518 (31.9)

Definition of AI as pertains to home healthcare	Using technologies that empower machines to carry out healthcare duties that demand human intelligence	407 (81.7)
	Using robots for delivering care at home	220 (44.2)
	A company creating computers	26 (5.2)

Examples of AI tools used for home healthcare	Wearable smart watches used to monitor vital signs, e.g., heart rate	799 (68.7)
	Health fitness trackers	634 (54.5)
	WhatsApp	369 (31.7)

Importance of using AI	Better response and tracking of infectious disease outbreak	711 (59.3)
	Early disease detection	733 (61.1)
	Early diagnosis and treatment	512 (42.7)
	Platform for discussing sports	177 (14.8)
	Help organize patient health information	238 (19.8)

Ways that AI can be used to control ERIDs	Enhance access to platforms for discussing health issues	653 (56.0)
	Use of wearable devices to track vital sign	736 (63.1)
	Use of mobile health apps can support decision about care and promote wide spread of infectious diseases	424 (36.4)
	Promote wild spread of infectious diseases	90 (7.7)

Heard about One Health	No	1372 (84.4)
	Yes	253 (15.6)

Definition of One Health	A treatment plan for diabetic patients	6 (0.4)
	An approach that recognizes that only human and animal health are connected	168 (10.3)
	An approach that recognizes that the health of humans, animals, and the environment are closely connected	67 (4.1)
	None of the above	12 (0.7)

Heard about Digital One Health	No	1390 (85.5)
	Yes	235 (14.5)

Definition of Digital One Health	An online shopping platform	6 (0.4)
	The use of digital technologies and data to better understand how human, animal, and plant health are interconnected	63 (3.9)
	Use of technology to understand human health only	158 (9.7)
	None of the above	8 (0.5)

Heard about digital health technologies	No	1149 (70.7)
	Yes	476 (29.3)

Examples of digital health technologies	ChatGPT	70 (15.3)
	Waspito	176 (38.5)
	Period calendar (PC)	285 (62.4)
	Wearable smart watches	210 (46.0)

Overall knowledge level	Good knowledge	166 (10.2%)
	Poor knowledge	1459 (89.8%)

**Table 4:** Association between sociodemographic variables and knowledge on AI and DOH among community members in the Buea, Limbe, Bonassama, and New-Bell Health Districts of Cameroon.

Variable	Category	Knowledge on AI–DOH		AOR	95% CI for AOR		*p* value
		Good (%)	Poor (%)		Lower	Upper	
Health district	Buea	89 (5.5)	368 (22.6)	3.724	2.176	6.371	0.001
	Limbe	31 (1.9)	411 (25.3)	1.387	0.758	2.537	0.289
	Bonassama	25 (1.5)	323 (19.9)	0.945	0.508	1.76	0.859
	New-Bell	21 (1.3)	357 (22.0)	1			
	Total	166 (10.2)	1459 (89.8)				

Sex	Male	85 (5.2)	653 (40.2)	1.603	1.129	2.276	0.008
	Female	81 (5.0)	806 (49.6)	1			
	Total	166 (10.2)	1459 (89.8)				

Level of education	Informal	8 (0.5)	93 (5.7)	0.947	0.410	2.188	0.898
	Primary	42 (2.6)	263 (16.2)	1.797	1.100	2.936	0.019
	Secondary	71 (4.4)	730 (44.9)	0.74	0.484	1.132	0.165
	Tertiary	45 (2.8)	373 (23.0)	1			
	Total	166 (10.2)	1459 (89.8)				

Main occupation	Employed in public sector	3 (0.2)	104 (6.4)	0.200	0.055	0.721	0.014
	Employed in private sector	16 (1.0)	205 (12.6)	0.586	0.278	1.238	0.161
	Self-employed	36 (2.2)	677 (41.7)	0.341	0.178	0.656	0.001
	Unemployed	14 (0.9)	105 (6.5)	1.105	0.505	2.419	0.802
	Student	81 (5.0)	201 (12.4)	2.741	1.483	5.066	0.001
	Jobless	16 (1.0)	167 (10.3)	1			
	Total	166 (10.2)	1459 (89.8)				

**Table 5:** Preparedness to utilize artificial intelligence and Digital One Health among community members in the Buea, Limbe, Bonassama, and New-Bell Health Districts of Cameroon.

Variables	Response	No. (%)
Prepared to use DHT	No	524 (32.2)
	Yes	1101 (67.8)
	Total	1625 (100.0)

Prepared to use AI	No	611 (37.6)
	Yes	1014 (62.4)
	Total	1625 (100.0)

Confidence in new DHT	No	667 (41.0)
	Yes	958 (59.0)
	Total	1625 (100.0)

Previous training on AI/DH tools	No	1276 (78.5)
	Yes	349 (21.5)
	Total	1625 (100.0)

Willing to take part in training	No	516 (31.8)
	Yes	1109 (68.2)
	Total	1625 (100.0)

Willing to receive care from HCP through AI	No	524 (32.2)
	Yes	1101 (67.8)
	Total	1625 (100.0)

Willing to recommend AI/DHT	No	503 (31.0)
	Yes	1122 (69.0)
	Total	1625 (100.0)

Wiling to use AI/DH tools during outbreaks	No	481 (29.6)
	Yes	1144 (70.4)
	Total	1625 (100.0)

Comfortable with AI interpretation	No	577 (35.5)
	Yes	1048 (64.5)
	Total	1625 (100.0)

Overall level of preparedness	High preparedness	941 (57.9%)
	Low preparedness	684 (42.1%)

**Table 6:** Sociodemographic factors independently associated with preparedness to use AI and DOH technologies for home healthcare for the control of emerging and reemerging infectious diseases among community members in the Buea, Limbe, Bonassama, and New-Bell Health Districts of Cameroon.

Variables	Category	Preparedness		AOR	95% CI for AOR		*p* value
		High (%)	Low (%)		Lower	Upper	
Level of education	Informal	44 (2.7)	57 (3.5)	0.476	0.295	0.767	0.002
	Primary	167 (10.3)	138 (8.5)	0.762	0.537	1.08	0.126
	Secondary	454 (27.9)	347 (21.4)	0.703	0.538	0.919	0.010
	Tertiary	276 (17.0)	142 (8.7)	1			
	Total	941 (57.9)	684 (42.1)				

Main occupation	Employed in public sector	47 (2.9)	60 (3.7)	0.665	0.401	1.104	0.115
	Employed in private sector	123 (7.6)	98 (6.0)	1.072	0.712	1.614	0.740
	Self-employed	394 (24.2)	319 (19.6)	1.14	0.806	1.614	0.459
	Unemployed	67 (4.1)	52 (3.2)	1.17	0.726	1.886	0.518
	Student	216 (13.3)	66 (4.1)	2.678	1.744	4.113	< 0.001
	Jobless	94 (5.8)	89 (5.5)	1			
	Total	941 (57.9)	684 (42.1)				

**Table 7:** Other factors independently associated with preparedness of community members to utilize AI and DOH for home healthcare delivery.

Variables	Response	Preparedness		AOR	95% CI for AOR		*p* value
		Low (%)	High (%)		Lower	Upper	
Knowledge about AI and DOH	Good	17 (1.0)	149 (9.2)	7.141	4.192	12.162	< 0.001
	Poor	667 (41.1)	792 (48.7)	1			
	Total	684 (42.1)	941 (57.9)				

Previous training on AI and DHTs	Yes	79 (4.9)	270 (16.6)	3.081	2.272	4.179	< 0.001
	No	605 (37.2)	671 (41.3)	1			
	Total	684 (42.1)	941 (57.9)				

Lack of human supervision in healthcare decision-making	Yes	310 (19.1)	466 (28.7)	0.783	0.621	0.988	0.039
	No	374 (23.0)	475 (29.2)	1			
	Total	684 (42.1)	941 (57.9)				

No access to AI technology	Yes	264 (16.3)	478 (29.4)	1.313	1.032	1.672	0.027
	No	420 (25.8)	463 (28.5)	1			
	Total	684 (42.1)	941 (57.9)				

Subsidizing cost	Yes	338 (20.8)	591 (36.4)	1.277	1.002	1.628	0.048
	No	346 (21.3)	350 (21.5)	1			
	Total	684 (42.1)	941 (57.9)				

Education/sensitization about AI	Yes	397 (24.4)	590 (36.3)	0.793	0.625	1.005	0.055
	No	287 (17.7)	351 (21.6)	1			
	Total	684 (42.1)	941 (57.9)				

Collaboration with healthcare providers	Yes	148 (9.1)	334 (20.5)	1.818	1.404	2.354	< 0.001
	No	536 (33.0)	607 (37.4)	1			
	Total	684 (42.1)	941 (57.9)				

Concerned about AI replacing healthcare providers	Yes	333 (20.5)	565 (34.8)	1.584	1.298	1.932	0.001
	No	351 (21.6)	376 (23.1)	1			
	Total	684 (42.1)	941 (57.9)				

Willingness of healthcare systems to integrate AI	Yes	148 (9.1)	443 (27.3)	3.382	2.67	4.284	< 0.001
	No	536 (33.0)	498 (30.6)	1			
	Total	684 (42.1)	941 (57.9)				

## Data Availability

The data used to support the findings of this study are available from the corresponding author upon request.

## Nomenclature

AI: Artificial intelligence
CI: Confidence interval
DOH: Digital One Health
EIDs: Emerging infectious diseases
ERIDs: Emerging and reemerging infectious diseases
IRB: Institutional Review Board
OH: One Health
RIDs: Reemerging infectious diseases
WHO: World Health Organization
LTR: Littoral Region
SWR: Southwest Region
